# Cannabinoid receptor-1 antagonism: a new perspective on treating a murine schistosomal liver fibrosis model

**DOI:** 10.1590/0074-02760190062

**Published:** 2019-08-05

**Authors:** Yasmine Amr Issa, Samar Nabil El Achy, Rasha Fadly Mady

**Affiliations:** 1 University of Alexandria, Alexandria Faculty of Medicine, Medical Biochemistry Department, Alexandria, Egypt; 2 University of Alexandria, Alexandria Faculty of Medicine, Pathology Department, Alexandria, Egypt; 3 University of Alexandria, Alexandria Faculty of Medicine, Medical Parasitology Department, Alexandria, Egypt

**Keywords:** cannabinoid receptor-1(CB1), schistosoma-associated liver fibrosis, rimonabant (SR141716), praziquantel, alpha smooth muscle actin

## Abstract

**BACKGROUND:**

Formation of schistosomal granulomata surrounding the ova can result in schistosomiasis-associated liver fibrosis (SSLF). The current standard of treatment is praziquantel (PZQ), which cannot effectively reverse SSLF. The role of the cannabinoid (CB) receptor family in liver fibrosis has recently been highlighted.

**OBJECTIVES:**

This study aimed to assess the therapeutic effect of CB1 receptor antagonism in reversing SSLF in a murine model of *Schistosoma mansoni* infection.

**METHODS:**

One hundred male Swiss albino mice were divided equally into five groups: healthy uninfected control (group I), infected control (group II), PZQ treated (group III), rimonabant (RIM) (SR141716, a CB1 receptor antagonist)-treated (group IV) and group V was treated with combined PZQ and RIM. Liver sections were obtained for histopathological examination, alpha-1 smooth muscle actin (α-SMA) immunostaining and assessment of CB1 receptor expression using real-time polymerase chain reaction (RT-PCR).

**FINDINGS:**

The most effective reduction in fibrotic marker levels and granuloma load was achieved by combined treatment with PZQ+RIM (group V): CB1 receptor expression (H = 26.612, p < 0.001), number of α-SMA-positive cells (F = 57.086, p < 0.001), % hepatic portal fibrosis (F = 42.849, p < 0.001) and number of granulomata (F = 69.088, p < 0.001).

**MAIN CONCLUSIONS:**

Combining PZQ with CB1 receptor antagonists yielded the best results in reversing SSLF. To our knowledge, this is the first study to test this regimen in *S. mansoni* infection.

Schistosomiasis is a common parasitic digenetic trematode infection. It is a very prevalent tropical disease in several developing countries like Egypt. More than 240 million people are infected worldwide and nearly triple that number (700 million) are at risk of acquiring the infection. Schistosomiasis is responsible for around 300,000 deaths annually in Africa alone.^1^

The histopathological hallmark of schistosomiasis is the development of epithelioid granulomata surrounding the ova with an eosinophilic-rich inflammatory infiltrate in the portal tracts of the liver. Fibrous scar tissue forms around the granulomata and eventually replaces them over time, resulting in fibrous expansion of the portal tracts and inducing hepatic fibrosis known as schistosomiasis-associated liver fibrosis (SSLF). SSLF is a consequence of massive deposition of extracellular matrix (ECM) in the periportal spaces, which blocks portal veins and leads to portal hypertension, portocaval shunting, cirrhosis, splenomegaly and gastrointestinal varices.^2^ ^)^

Two major events that generally characterise liver fibrosis are activation and proliferation of hepatic stellate cells (HSCs) and an increase in ECM deposition, especially collagen type I.^3^ Increasing evidence has highlighted the role of activated myofibroblasts residing in the portal tracts encircling bile ducts and ductules and portal vein and hepatic artery radicals in the pathogenesis of portal fibrosis.^4^ HSCs and myofibroblasts derived from different cell populations express alpha-1 smooth muscle actin (α-SMA) and synthesise fibrogenic cytokines [transforming growth factor (TGF-β1)], growth factors, chemokines, fibrosis components and inhibitors of matrix degradation.^5^ ^,^ ^6^ ^)^

Despite the presence of efficacious schistosomicides like praziquantel (PZQ), it has been demonstrated that the granulomatous inflammatory reactions and hepatic fibrosis continue aggressively even with efficient treatment.^7^ To date, several attempts have been made with other treatment modalities either alone or in combination with PZQ. However, there has been scant treatment for direct effect on fibrosis-forming cells in the case of schistosomiasis.^8^

It was recently shown that neuro-humoral signalling plays a role in HSC responses. Particularly, the endogenous lipidic cannabinoid (CB) ligands and their receptors, CB1 and CB2, have emerged as potent mediators of hepatic steatosis, stellate cell activation and hepatic fibrosis.^9^ In addition, CBs provoke the hemodynamic alterations associated with advanced liver disease.^3^ The two receptors, CB1 and CB2, exert opposing effects: CB1 activates the fibrogenic pathway and CB2 is antifibrotic.^10^ Although CB1 receptors are the most abundant receptors in the mammalian brain, they are also expressed at lower levels in a large number of peripheral tissues like various liver cell types. Based on these findings, antagonism of CB1 signalling in HSCs has emerged as a promising antifibrotic strategy.^6^

Here, we assessed the therapeutic effect of antagonising CB1 receptors (using CB1 receptor antagonist, rimonabant (RIM) (SR141716) in reversing SSLF for the first time in a murine model of *Schistosoma mansoni* infection.

## MATERIALS AND METHODS

*Animals, parasite and drugs -* This study was carried out in the Parasitology, Medical Biochemistry and Pathology departments of Alexandria Faculty of Medicine, Alexandria University, Egypt. 

The study used 100 male Swiss albino mice (four to six weeks old and about 20-25 grams weight) purchased from the animal house, Medical Parasitology Department, Faculty of Medicine, Alexandria University, Alexandria, Egypt. The mice were given tap water and a balanced ad libitum diet. 

Ethical committee rules regarding animal housing and sacrifice were followed. All animal studies were approved by the local government based on national regulations for animal experimentation.

*S. mansoni* cercariae were shed from infected *Biomphalaria alexandrina* snails purchased from the Schistosoma Biological Supply Program (SBSP) in Theodor Bilharz Research Institute (TBRI), Imbaba, Giza, Egypt. They were used for infection of 80 mice with a dose of 100 freshly shed cercariae/mouse using the tail immersion technique.^11^

Two drugs were used: PZQ (Distocide) EIPICO, Cairo, Egypt, was purchased from a local pharmacy and RIM hydrochloride (SR141716, RIM), a CB-1 receptor antagonist, was purchased from Sigma Aldrich (catalogue number SML0800). 

*Experimental design -* Mice were divided into five groups of 20 mice each; normal uninfected control (group I), infected control (group II), PZQ-treated (group III), RIM-treated (group IV) and group V treated with combined PZQ and RIM. All treatment regimens commenced at week 8 post-infection (pi). The timing was chosen because liver fibrosis is expected to be successfully established eight weeks after infection.^12^ Drugs were administered once daily for two weeks.

PZQ was given to groups III and V at a dose of 300 mg/kg and RIM was given to groups IV and V at a dose of 10 mg/kg dissolved in 1 mL/kg of saline solution with a drop of Tween 80.^13^

Mice were sacrificed at week 10 pi. Liver specimens from sacrificed mice were divided. Part of each specimen was conserved in RNA later stabilisation reagent (Qiagen, USA, catalogue no. 76104) and stored at -80º C for later CB1 receptor expression assessment. The other part was placed in 10% buffered formalin for histopathological assessment.

*Assessment of CB1 receptor expression using real-time polymerase chain reaction (RT-PCR) -* On the day of the assay, 30 mg of liver tissue sections were transferred to RNase-free round-bottomed tubes on ice to be homogenised (0.6 mL of freshly prepared Qiazol lysis reagent) (Qiagen, Germantown, MD, USA; cat. no. 79306) containing 1% of 2-mercaptoethanol was added to each tissue sample. Homogenisation was performed using a rotor-stator according to the manufacturer’s protocol. Total RNA was extracted and purified using PureLink^®^ RNA Mini Kit (Invitrogen, Waltham, MA, USA; cat. no 12183018A). Concentration of total RNA was estimated using Nanodrop. 

Purified RNA was reverse transcribed using Applied Biosystems (Waltham, MA, USA) High-Capacity cDNA Reverse Transcription Kits (cat. no. 4374966). Briefly, 2 μg of total RNA was used per 20 μL reaction. The thermal cycler was programmed as follows: 25ºC for 10 min, 37ºC for 120 min, 85ºC for 5 min and 4ºC until the removal of samples. A minus reverse transcription control was added in all experiments to rule out DNA contamination. Complementary DNA (cDNA) was stored at -20ºC until CB1 receptor expression assessment. RT-qPCR was performed using an Applied Biosystems Step-one Real-time system. For each sample, 1 µL of Taqman CB1 gene expression assay reagent [Thermo Fischer (Waltham, MA, USA) scientific as­say no. Hs01038522_s1, cat. no. 4331182] was added to 10 µL of Taqman master mix and 5 µL of RNAse free H_2_O. Four microliters of the cDNA sample was added to complete the total volume to 20 µL. GAPDH (Taqman GAPDH control reagent, Thermo Fischer, cat. no. 402869) was used as the endogenous reference gene for data normalisation. RT-PCR settings were as follows: an initial two-minute hold cycle at 50^o^C, 10 min hold 95^o^C and 40 cycles of 15 sec at 95^o^C and 1 min at 60^o^C.

*Histopathological studies -* Liver biopsy specimens were fixed in 10% buffered formaldehyde for 24 h and processed routinely. The tissue samples were embedded into paraffin blocks and sectioned into 4 µm thick sections for staining with haematoxylin and eosin (H&E) and Masson’s Trichrome stain at three non-continuous levels. 

Each sample was analysed histologically for the number of schistosomal granulomata, the constituent cells of the granulomata and presence of epithelioid and inflammatory cell infiltrates or myofibroblasts and fibrosis. Lobular necro-inflammatory activity was also assessed. 

*Immunohistochemical staining -* Two consecutive four-micron-thick tissue sections of representative paraffin blocks were cut and mounted onto positively charged slides. Heat-induced antigen retrieval using citrate buffer at pH 6 was performed. Immunostaining using polyclonal α-SMA (Invitrogen #PA1-37024) primary antibody at a dilution of (1:100) was performed, followed by Streptavidin-HRP Conjugate (Thermo Scientific # D22187) and developed using DAB chromogen with Meyer’s haematoxylin as a counterstain to assess myofibroblastic cells. 

Quantitative assessment of the α-SMA-positive portal myofibroblastic cells showing cytoplasmic positivity was performed using a mean value for the number of positive cells in ten high power fields (HPF).

*Semi-quantifying the fibrosis extent -* Quantitative analysis of the fibrosis area within the portal tracts of the liver was performed on the Masson’s Trichrome stained sections at three non-continuous sections using a morphometric analysis software (Olympus BX41, Tokyo, Japan). 

*Ethics -* Ethical committee rules were followed regarding animal housing and sacrificing. All animal studies were approved by the local government based on national regulations for animal experimentation.

## RESULTS

*Statistical analysis of the data -* Data were analysed using IBM SPSS software package version 20.0 (IBM Corp, Armonk, NY, USA). Qualitative data were described by number and percent. The Kolmogorov-Smirnov test was used to verify the distribution normality. Quantitative data were described using mean, standard deviation and median. Significance of the obtained results was judged at the 5% confidence level. F-test [analysis of variance (ANOVA)] was used for normally distributed quantitative variables to compare between more than two groups and post-hoc test (Tukey) was used for pairwise comparisons. Kruskal Wallis test was used for abnormally distributed quantitative variables to compare between more than two studied groups and post-hoc (Dunn’s multiple comparisons test) was used for pairwise comparisons. The Pearson coefficient was used to correlate between two normally distributed quantitative variables.

*CB1 expression, fibrotic area extent, average number of α-SMA positive cells and average number of granulomata (* Table *) -* We compared the uninfected control group (I) to the infected control group (II) to verify the increased CB1 expression, increased SMA-expressing cells, fibrosis extent and granulomata formation. All parameters were significantly increased in infected mice (p < 0.001 in all parameters).

**TABLE:** Comparison between the four studied groups according to different parameters

RQ	Control (group II) (n = 20)	PZQ (group III) (n = 20)	RIM (group IV) (n = 20)	PZQ+RIM (group V) (n = 20)	Test of sig.	p-value
	1 (1-1)	0.31 (0.07-0.9)	0.07 (0.01-0.5)	0.02 (0.06-0.1)	H = 30.966^*^	< 0.0001^*^
p_control_		0.069	< 0.001^*^	< 0.001^*^		
Statistical significance bet. groups		p_1_ = 0.031^*^, p_2_ = 0.001^*^, p_3_ = 0.254				
Fibrosis (%)	86.7 ± 4.1	57.3 ± 15.4	35 ± 12	21.8 ± 11.2	F = 42.849^*^	< 0.001^*^
p_control_		< 0.001^*^	< 0.001^*^	< 0.001^*^		
Sig. bet. groups		p_1_ = 0.001^*^, p_2_ < 0.001^*^, p_3_ = 0.081				
Granulomata	17.5 ± 1	12.4 ± 1.5	14.9 ± 1.2	8.3 ± 1.6	F = 69.088^*^	< 0.001^*^
p_control_		< 0.001^*^	0.005^*^	< 0.001^*^		
Sig. bet. groups		p_1_ = 0.001^*^, p_2_ < 0.001^*^, p_3_ < 0.001^*^				
Average number of α-SMA-positive cells	74.7 ± 7.1	47.1 ± 15.1	29.2 ± 5.4	14.9 ± 5.5	F = 57.086^*^	< 0.001^*^
p_control_		< 0.001^*^	< 0.001^*^	< 0.001^*^		
Sig. bet. groups		p_1_ = 0.001^*^, p_2_ < 0.001^*^, p_3_ = 0.008^*^				

Normally quantitative data was expressed as mean ± standard deviation and compared using analysis of (ANOVA) test, and post-hoc test (Tukey) for pairwise comparisons. While abnormally distributed data was expressed using median (min.-max.) and was compared using Kruskal Wallis test and post-hoc (Dunn’s multiple comparisons test) for pairwise comparisons. p1: p-value for comparing between praziquantel (PZQ) and rimonabant (RIM); p2: p-value for comparing between PZQ and PZQ+RIM; p3: p-value for comparing between RIM and PZQ+RIM; pcontrol: p-value for comparing between control group with each other group; RQ: relative quantification; Sig: significance; α-SMA: alpha smooth muscle actin. *: statistically significant at p ≤ 0.05.

To show the efficacy of different treatment modalities in reducing fibrotic markers, all treated groups were compared to each other and the control group II. Results showed that CB1 expression was mostly reduced in the combined treatment RIM+PZQ group [group V, median relative quantification (RQ) = 0.02], followed by group IV treated by RIM (median RQ = 0 .07). Group III treated with PZQ showed the smallest reduction in CB1 expression (median RQ = 0.31). Comparing all groups showed statistically significant differences in CB1 expression (H = 30.966, p < 0.0001).

The same pattern was followed regarding the extent of fibrotic areas. The mean fibrotic areas were measured in three non-consecutive sections of liver specimens and expressed as a percentage of the whole area of liver tissue. The mean fibrosis percentage was 21.8% in group V, 35% in group IV and 57.3% in group III compared to 86.7% in control group II. Comparing all the groups showed statistically significant differences in fibrosis extent (F = 42.849, p < 0.001).

Logically, the same applied to the mean number of α-SMA expressing cells, which was 14.9 in group V, 29.2 in group IV and 47.1 in group III compared to 74.7 in control group II. Comparing all the groups showed statistically significant differences in the average number of cells expressing SMA (F = 57.086, p < 0.001).

 The pattern was slightly different regarding the average number of granulomata, where group V showed the greatest reduction in granulomata number (mean = 8.3), followed by group III (mean = 12.4) and group IV (mean = 14.9) compared to the control group II (mean = 17.5). Comparing all the groups showed statistically significant differences in the average number of granulomata (F = 69.088, p < 0.001)

**Fig. 1:**
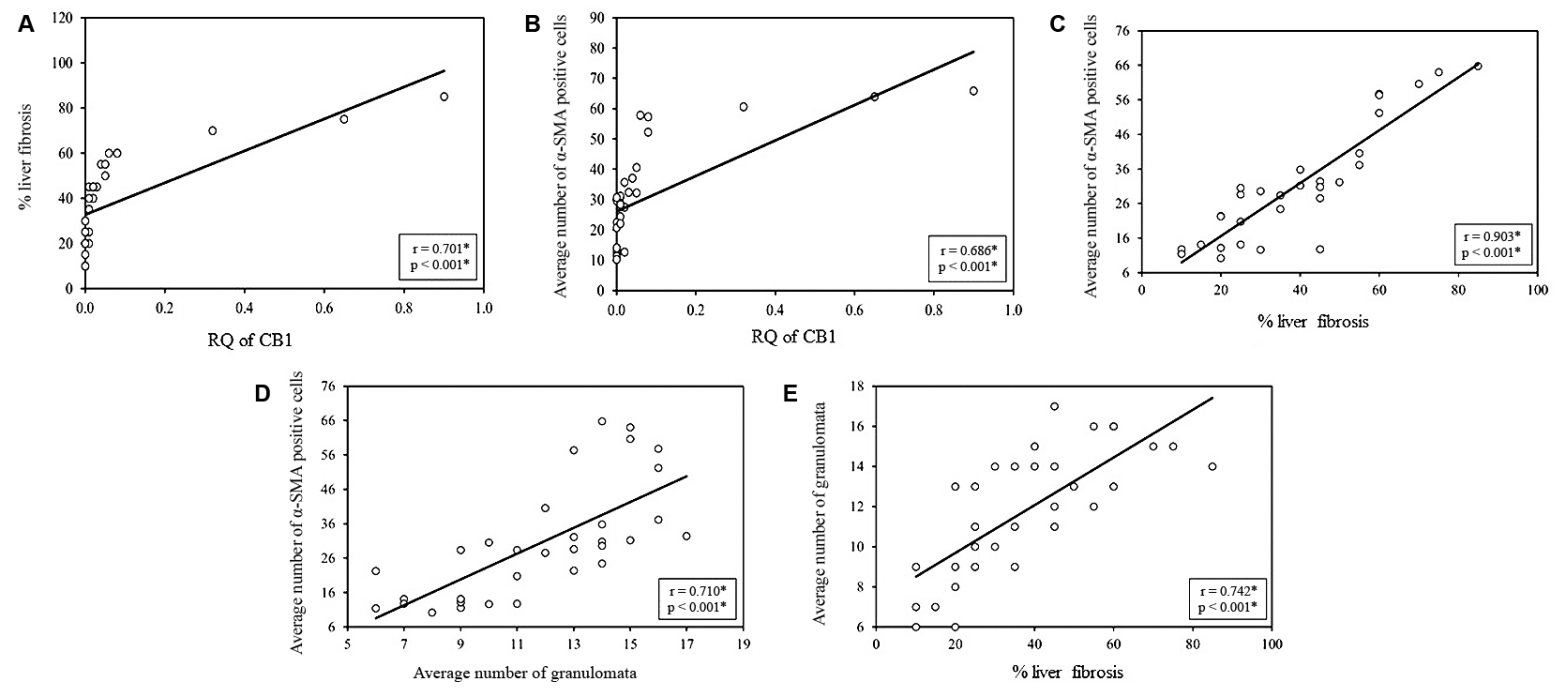
correlations between different studied parameters. CB1: cannabinoid receptor 1; RQ: relative quantification; α-SMA: alpha smooth muscle actin. *: statistical significance at a p ≤ 0.05.

CB1 expression was found to significantly correlate to each of extent of fibrosis measurement (r = 0.701, p < 0.001) (Fig. 1A) and the number of SMA-expressing cells (r = 0.686, p < 0.001) (Fig. 1B), but not significantly to the number of granulomata (r = 0.318, p = 0.076). The average number of SMA-expressing cells was significantly correlated to fibrosis extent (r = 0.903, p < 0.001) (Fig. 1C) and average number of granulomata (r = 0.710, p < 0.001) (Fig. 1D). Naturally, the average number of granulomata was significantly correlated to the fibrosis extent (r = 0.742, p < 0.001) (Fig. 1E)

**Fig. 2:**
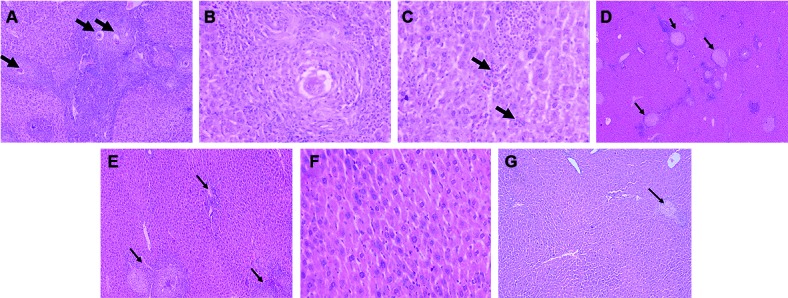
histopathological features of liver tissues in control infected mice (II) and treated mouse groups (III, VI, V). A: a photomicrograph of a control infected mouse liver showing abundant schistosomal eosinophilic granulomas expanding the portal tracts (shistosomal ova; arrows). The portal tracts are expanded and heavily infiltrated by inflammatory infiltrate, predominantly eosinophils, with spill-over into the lobules haematoxylin and eosin (H&E), 100X; B: a photomicrograph of a control infected mouse liver showing a higher power view of hyalinised schistosomal ovum surrounded by a granulomatous reaction composed of epithelioid cells, lymphocytes, plasma cells and eosinophils (H&E, 400X); C: a photomicrograph of a control infected mouse liver showing feathery degeneration of the hepatocytes and intralobular inflammatory cellular infiltrates with focal hepatocyte necrosis (arrow) (H&E, 400X); D: a photomicrograph of a PZQ-treated mouse (group III) liver showing abundant schistosomal granulomata within the portal tracts exhibiting reduced expansion of the portal tracts by inflammatory infiltrates (arrows) (H&E 40X); E: a photomicrograph of a rimonabant (RIM)-treated mouse (group IV) liver showing schistosomal eosinophil-rich epithelioid granulomata with mild to moderate inflammation in the portal tracts (arrows) (H&E, 100X); F: a photomicrograph of a RIM-treated mouse (group IV) liver showing moderate to marked Kupffer cell hyperplasia in the hepatic lobules. Minimal hepatocyte injury is noted (H&E, 400X); G: a photomicrograph of a combination therapy mouse (group V) showing fewer and smaller granulomata (arrow) in the portal tract with minimal inflammatory infiltrates and fibrosis (H&E, 100X).

**Fig. 3:**
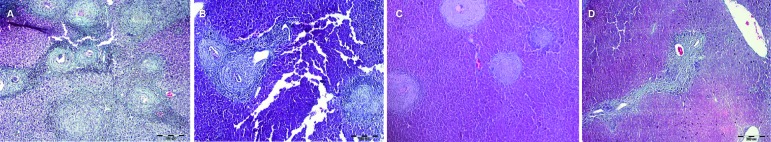
trichrome staining of the liver specimens of control and all of the three treated mice groups III, IV, V. A: a photomicrograph of a control infected mouse liver (group II) showing massive expansion of the portal tracts by fibrous tissue as well as the shistosomal granulomata (100X); B: a photomicrograph of a praziquantel (PZQ) treated mouse liver (group III) showing moderate fibrous expansion of the portal tracts with the mere presence of granulomata (100X); C: a photomicrograph of a rimonabant (RIM)-treated mouse liver (group IV) showing minimal fibrous expansion of the portal tracts with minimal fibrosis around the schistosomal granulomas (100X); D: a photomicrograph of a combination therapy mouse liver (group V) minimal fibrosis within the portal tracts with small granulomata (100X).

*Histopathologic examination -* Control infected mice (group II) - Microscopic examination of the control mouse livers revealed marked expansion of the portal tracts by schistosomal granulomata centred around the schistosomal ova. The granulomata were composed of epithelioid cells, lymphocytes, plasma cells and eosinophils and surrounded by an outer zone of fibroblastic cells (Fig. 2A, B). The portal tracts were markedly expanded by heavy inflammatory infiltrates with spill-over into the hepatic lobules predominated by eosinophils. Hepatocytes revealed evidence of feathery degeneration and foci of hepatocyte necrosis with intralobular aggregates of lymphocytes and eosinophils (Fig. 2C). Sinusoidal dilation and intrasinusoidal lymphocytes were also evident. Kupffer cells were not evidently hyperplastic. Trichrome-stained sections revealed the notable expansion of portal tracts by fibrous tissue (Fig. 3A).

PZQ-treated mice livers (group III) - Examination revealed moderate expansion of the portal tracts by schistosomal granulomata with reduced amounts of inflammatory cells surrounding the granulomata, but portal fibroblastic cell proliferation was evident (Fig. 2D). Hepatocytes did not reveal morphologic signs of injury and only a moderate increase in the number of Kupffer cells was noted. Trichrome-stained sections revealed moderate expansion of the portal tracts by fibrous tissue (Fig. 3B). 

RIM-treated mice livers (group IV) - Examination revealed a mild to moderate expansion of portal tracts by schistosomal granulomata comprising mainly inflammatory cellular infiltrate and minimal portal myofibroblastic proliferation (Fig. 2E). Trichrome-stained slides showed minimal fibroblastic expansion of the portal tracts in the study group (Fig. 3C). The hepatocyte lobules revealed unremarkable morphological changes in the hepatocytes, but marked Kupffer cell hyperplasia was noted (Fig. 2F). 

PZQ+RIM treated mice livers (group V) - Examination revealed marked diminution of portal tract expansion by the schistosomal granulomata (Fig. 3D), a reduced number of inflammatory cells and reduced fibrosis area (Fig. 2G). 

**Fig. 4:**
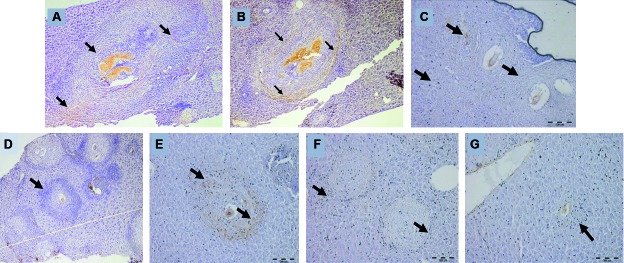
alpha smooth muscle actin (α-SMA) immunostaining of the liver specimens of control group (II) treated mouse groups (III, IV, V). A: a photomicrograph of a control infected mouse rat liver showing positive staining of portal myofibroblastic cells for α-SMA immunostain around the portal tracts and the schistosomal granulomata (α-SMA, 40X); B: a higher power view of the previous slide showing α-SMA positive staining portal myofibroblastic cells surrounding the portal tracts and schistosomal granulomata (arrows) (α-SMA, 100X); C: a high power view of a schistosomal granuloma in a control infected mouse liver showing positively stained anti-SMA portal myofibroblastic cells (arrows) (α-SMA, 400X); D: a photomicrograph of a praziquantel (PZQ)-treated mouse (group III) liver showing positively stained anti-SMA myofibroblastic cells in the portal tracts (α-SMA, 100X); E: a high power view of a schistosomal granuloma in a PZQ-treated mouse (group III) liver showing positively stained anti-SMA hepatic stellate cells (arrows) (400X); F: a photomicrograph of rimonabant (RIM)-treated mouse (group IV) liver showing a few positively stained anti-SMA myofibroblastic cells (arrows) surrounding the granulomata (α-SMA, 200X); G: a photomicrograph of a combination therapy mouse liver (group V) showing scant SMA positive stained cells in the portal tracts (α-SMA, 200X).

*Alpha-smooth muscle actin immunohistochemistry staining -* Examination of the study groups revealed prominent cytoplasmic staining of the portal myofibroblastic cells surrounding the schistosomal granulomata and within the portal tracts in the infected control group II (Fig. 4A-C). The number of α-SMA positive myofibroblastic cells was significantly reduced in the combined PZQ+RIM-treated group (Fig. 4G), followed by the RIM treated group (Fig. 4F) compared to controls. The group treated with only PZQ did not show a significant reduction in the number of α-SMA positive cells (Fig. 4D, E).

## DISCUSSION

In addition to being a neglected tropical disease, schistosomiasis is second only to malaria as a major parasitic disease. Although chemotherapy can successfully treat schistosomiasis, few drugs can reverse hepatic fibrosis. Patients with advanced SSLF can only undergo surgery for splenomegaly or oesophageal varices to avoid fatal complications. Consequently, the importance of finding a new effective antifibrotic drug has emerged. 

Based on the known role of CB1 receptors in liver fibrosis acceleration, this was conducted to assess the role of RIM as a CB1 receptor antagonist in a murine model of *S. mansoni* hepatic fibrosis. The efficacy of RIM was tested both alone and in combination with PZQ. To our knowledge, this is the first study to attempt this regimen for *S. mansoni* infection.

Here, significant CB1 receptor overexpression was demonstrated in *S. mansoni*-infected control mice with fibrotic livers compared to healthy control mice. This was associated with significantly increased rate of fibrosis, average number of α-SMA-expressing cells and granulomata formation. Our results agree with those obtained by Liu et al.,^14^ who used murine models of *Schistosoma japonicum* infection and demonstrated increased CB1 and CB2 expression in liver tissues around the schistosomal granuloma tubercles and lymphocytes during liver injury.

As an explanation for such results, it is known that chronic liver injury healing is usually prolonged and dysregulated. Imbalance between ECM synthesis and degradation occurs first and it is followed by fibrosis. TGF-β favours the transition of quiescent HSCs to myofibroblast-like cells, stimulates the synthesis of ECM proteins and inhibits their degradation.^6^ ^,^ ^15^ Portal myofibroblasts and activated HSCs both express α-SMA and the average number of α-SMA cells increases in the fibrotic liver.^16^

Regarding increased expression of CB1 receptors in fibrotic liver, studies showed that the CB1 receptor was expressed in normal liver,^17^ but the basal expression in the adult healthy liver is low.^5^

Although CBs have long been known for their psychoactive effects, the role they play in hepatic fibrosis has emerged with the characterisation of the endocannabinoid system (ECS).^10^ ECS includes at least two specific G-protein coupled receptors (CB1 and CB2), their endogenous lipidic ligands {endocannabinoids like arachidonoyl ethanolamide [anandamide (AEA)] and [2-arachidonoylglycerol (2-AG)]} and the enzymes involved in their synthesis and degradation.^9^

Several conditions characterised by chronic liver damage, including hepatic fibrosis, are associated with upregulation of both CB1 and CB2 hepatic receptors.^18^ CB1 receptors were found to be significantly upregulated in the vascular endothelium and in myofibroblasts in fibrotic bands of cirrhotic livers of humans and rodents.^5^ ^,^ ^17^

CB2 receptors are mainly expressed by immune cells.^10^ ^,^ ^19^

Studies showed that the activation of these receptors exerts opposing effects on the fibrogenic process: CB1 is pro-fibrogenic and CB2 is anti-fibrogenic.^18^ The opposing effects of both receptors can be attributed to their different cellular distributions in the injured liver. As mentioned above, CB1 is expressed in hepatocytes, HSCs and vascular endothelium, so it may alter the response to injury in these cell types.^20^ On the other hand, it is thought that CB2 may mediate its anti-fibrotic actions through anti-inflammatory signals since it was shown to be mainly expressed in monocytic cell types in patients with non-viral causes of hepatic fibrosis.^19^ It seems that the induction of ECS at the level of endocannabinoids and their receptors affect the course of liver fibrogenesis via a balance between the two complementary mechanisms exerted by CB1 and CB2 receptors.^21^

Agreeing with our results, Dai et al.^10^ reported that the liver biopsies of chronic hepatitis B patients showed both CB1 receptor overexpression and increased α-SMA count in fibrotic livers. Teixeira-Clerc et al.^5^ used biopsies of human chronic liver disease and they reported that cultured human and mouse liver fibrogenic cells and activated HSCs also expressed CB1 receptors. Wang et al.^7^ also demonstrated similar findings in cultured HSCs from *S. japonicum*-infected mice and they attributed increased CB1 receptor expression in fibrotic liver to NADPH oxidase redox regulation. Patsenker et al.^22^ demonstrated that CB1 receptor overexpression is restricted only to fibrotic areas associated with alcoholic liver cirrhosis. They also showed that CB1 receptor knockout mice were highly resistant to developing alcoholic liver fibrosis, underscoring the role that the CB1 receptor plays in fibrogenesis.

As for the efficacy of antagonising the CB1 receptor, as an attempt to reverse hepatic fibrosis, our study showed that in infected mice CB1 receptor expression, fibrotic changes and the number of cells positive for α-SMA were significantly reduced after treating with a RIM+PZQ combination. Similar results with a mildly lower efficacy (but still statistically significant) were seen in the RIM-treated group. In the PZQ treated group, PZQ alone decreased the number of granulomata in the liver. However, a slight non-significant effect was noticed on fibrotic markers. This is attributed to the successful use of PZQ as an anti-schistosomal drug that reduces the worm load, egg deposition and granuloma formation, even though it does not effectively reduce fibrosis.

Previous studies^5^ ^,^ ^13^ demonstrated that blocking the CB1 receptor by pharmacological antagonists like RIM (SR141716A) or genetic inactivation like in Cnr1-/- mice limited the progression of hepatic fibrosis whether the fibrosis was induced by bile duct ligation, CCL4 or thioacetamide. However, those studies did not assess fibrosis caused by parasitic infestations.^5^ This is believed to be due to inhibition of the activation and growth of hepatic myofibroblasts and reduced hepatic TGF-β1 levels. CB1-/- mice also showed elevated MMP-9 and -13 levels, indicating a shift toward enhanced fibrolysis. It is note-worthy that blocking the CB1-receptor in healthy animals showed minimal effects, suggesting that ECS has only limited tonic activity under normal physiological conditions.^23^ In a study by Gary-Bobo et al.,^24^ RIM treatment for eight weeks reversed hepatic steatosis in obese rats to levels comparable to that in their lean counterparts. In addition, treatment resulted in lower levels of liver injury enzyme markers like alanine transaminase, alkaline phosphatase and gamma glutamyltransferase. In the same study, levels of the pro-inflammatory marker tumour necrosis factor alpha (TNF-α) were also decreased. 

The mechanisms by which endocannabinoids and their receptors modulate fibrosis seem to be via cell death and proliferation regulation and immune response modulation.^21^ The disappearance of activated HSCs and myofibroblasts precedes the resolution of fibrosis, so it is possible that mediators within the liver can either induce death of HSCs and myofibroblasts or reverse their activation. Research showed that resolution of liver fibrosis is usually accompanied by HSC apoptosis, suggesting that selective induction of HSC death can help reduce fibrosis.^25^

It is interesting that in some cells like neuronal cells, the CB1 receptor can activate cytoprotective pathways like the phosphatidylinositol 3-kinase pathway. Therefore, blocking the CB1-receptor should eliminate this anti-apoptotic effect and ultimately lead to cell apoptosis. This is interesting since HSCs share many molecular markers and features with neuronal cells.^21^ In keeping with such results, Dai et al.^26^ demonstrated that administration of RIM reduced HSC proliferation and increased HSC apoptosis in HSC-T6 cell culture. RIM seems to down-regulate CB1 receptor mRNA expression. In the same study, cell cycle analysis showed a decrease in G2/M phase cells and an increase in G0/G1 phase cells in HSC-T6 cells treated with RIM. Additionally, apoptosis was increased based on the increase in caspase-3 protein expression. Moreover, collagen secretion was decreased and expression of phosphorylated focal adhesion kinase (FAK)/protein kinase B (AKR) and Raf/extracellular-signal-regulated kinase was inhibited.^27^ The two kinases govern fibrogenesis by regulating protein synthesis, transcription of pro-fibrogenic genes and controlling the cell cycle, cell proliferation and apoptosis. They are known to be mediators of anti-apoptotic and pro-proliferative effects in HSCs and myofibroblasts. Gallotta et al.^28^ confirmed that RIM can induce apoptosis via both caspase-dependent and independent pathways. 

The CB1 receptor’s cytoprotective effect was also documented in hepatic myofibroblasts, where it appears to prevent spontaneous agonist-independent cell death. This was seen even in the absence of EC ligands, which raised the possibility that CB1 might possess a signalling capacity on its own even in the absence of ligand activation.^5^ In the same study, CB1 receptor-deficient myofibroblasts were found to express lower baseline ERK and Akt activation.

Patsenker et al.^22^ also concluded that use of RIM in HSC culture resulted in DNA synthesis inhibition approximately fourfold after adding both 5 and 10 μmol/L of RIM and it downregulated α-SMA, procollagen α-1 and tissue inhibitor of metalloproteinase-1(TIMP-1) mRNA expression by more than 50% when the same dose of RIM was used. Lower doses below 1 μmol/L showed no effect on cellular functions. The same study drew attention to the importance of the RIM dose on cytotoxic effects. They concluded that RIM possesses potent anti-fibrotic properties at 5 μmol/L without causing significant cell death, but cytotoxic effects were observed at higher doses. 

To our knowledge, this current study is the first to study CB1 receptor expression and antagonism in an *S. mansoni*-infected murine model of SSLF. Although this study showed that RIM is highly effective in reversing SSLF, there still is a limitation. Despite that, mice treated with RIM showed no signs of altered behaviour nor significant weight loss. However, meta-analysis of nine clinical trials concluded that a small number of subjects treated with 20 mg per day of RIM showed signs of depressive disorders.^29^ This led to the development of a newer generation of peripheral CB1 receptor antagonists like AM6545^30^ and JD5037.^31^ Unlike RIM, these peripheral antagonists are less lipid-soluble and less liable to penetrate the blood brain barrier, reducing the central neuropsychiatric adverse effects. They are currently in the pre-clinical stage of development. Cinar et al.^32^ used a hybrid CB1R (ibipinabant)/iNOS antagonist called MRI-1867. This compound could reverse fibrosis in a mouse model of fibrosis induced by bile duct ligation. 

Further larger scale studies of schistosomal hepatic fibrosis and other causes of liver fibrosis are required to validate the efficacy of peripheral CB1 receptor antagonists in reversing liver fibrosis.

## CONCLUSION

Pharmacological antagonism of CB1 receptors using RIM is an effective treatment for reversing SSLF, especially when combined with the classical schistosomicide PZQ. Due to the reported neuropsychiatric adverse effects of the central CB1 receptor blockers, further trials with the recently developed peripheral CB1 receptor antagonists are required to limit possible adverse effects. Studies using CB1 receptor blockers on other causes of liver fibrosis are warranted to elucidate effectiveness in reversing fibrosis to pave the way for better liver disease management.
